# Prediction of Mortar Compressive Strength Based on Modern Minor-Destructive Tests

**DOI:** 10.3390/ma16062402

**Published:** 2023-03-17

**Authors:** Dawid Łątka

**Affiliations:** Faculty of Civil Engineering, Cracow University of Technology, Warszawska 24, 31-155 Kraków, Poland; dawid.latka@pk.edu.pl; Tel.: +48-12-628-23-96

**Keywords:** masonry, mortar compressive strength, minor-destructive testing, in situ diagnostic, penetrometric test, double punch test, structural health monitoring, mortar curing, empirical equations

## Abstract

The crucial task of the diagnosis of an existing masonry structure is to assess the current values of the mechanical parameters of the materials from which the structure was erected—usually bricks and mortar. The article presents the results of minor-destructive tests carried out on bed joints of three-brick-masonry prisms prepared in the laboratory. Three types of mortars used in the masonry were tested, which differ by the type and amount of binder. In order to determine mortar compression strength, three modern diagnostic methods were used: double punch test (DPT), standard penetrometric test (PT) and torque penetrometric test (TPT). Tests were carried out after 4, 12 and 90 weeks. The mortar strength determined in each of these tests was compared with the mortar reference strength determined on the beam specimen according to the methodology given in EN 1015-11. The results of the conducted tests confirmed the high usefulness of all three diagnostic methods. However, limitations in the application of the PT test were noticed—only lime mortars and weak cement–lime mortars can be tested with this method. In the case of mortars with an increased amount of cement binder, the impact energy is too low to estimate the compressive strength of the mortar in the brick wall joint. Technical limitations in the use of TPT and DPT tests were also indicated—weak lime mortars with low cohesion do not allow for obtaining reliable results. It was shown that DPT results strongly depend on two factors, specimen slenderness and mortar strength. Due to this fact, simple non-parameter conversion from mortar compressive strength according to the DPT test into mortar reference strength may lead to significant overestimation. As the results show, in newly built masonry, proper selection of diagnostic method is crucial due to the strong dependence of mortar curing dynamics on its location in the joint. This paper helps to match diagnostic techniques with the condition and type of mortar in the existing structure.

## 1. Introduction

Very often, when assessing the possibility of increasing the live loads in a building, its expansion or even periodic inspection of its technical condition, the mechanical parameters of the structure should be determined. The most appropriate way is to measure them directly during destructive tests (DT) [1]. Due to the characteristic feature of historical masonry—which is a large heterogeneity of the structure—the number of specimens to be tested and their size must be large. However, the cutting out of masonry prisms is rarely encountered due to the fact that it damages the structure considerably, is expensive and often difficult to implement due to the low fragmentation resistance of the specimen taken. A modern alternative is the flat-jacks test, which is conducted in situ. During the test, the actual stress–strain relationship of the wall is recorded. Despite its versatility and numerous advantages, the use of this method is strongly limited. The necessity to have specialized equipment and personnel with extensive experience in interpreting the obtained results are the most important limitations, also confirmed by the author’s own experience [2]. A cylindrical specimen of 100–150 mm diameter obtained as boreholes are an increasingly popular alternative [3]. However, due to technical problems with keeping the specimens undisturbed during the extraction from the wall and the unfavorable influence of water cooling the blade during the drilling, this method requires the researcher to have prior practice.

An easier, and thus often used, method of estimating the compressive strength of a brick wall (as a leading mechanical parameter) is to determine the compressive strength of mortar and bricks separately. The desired compressive strength of the wall is determined on the basis of correlations obtained empirically and published in the literature. This method is especially sanctioned in modern design codes such as EN 1996-1-1 [4]. The strength of bricks is determined in a very simple way directly on whole bricks taken from the structure or on their halves. In order to reduce the number of bricks to be taken for the study, small cylindrical or cube samples are also used; however, taking into account the anisotropy of the bricks and the influence of scale effect on the results is needed [3,5]. It is incomparably more difficult to estimate the compressive strength of the mortar in the existing structure, which results from two basic factors: small joint thicknesses (usually below 20 mm) and degradation of the surface layer of mortar in the joint. The dimensions of the mortar specimen for strength tests required by EN 1015-11 [6] should be 4 × 4 × 8 cm^3^, which excludes the application of the research methodology of this standard in the case of existing buildings. Sometimes few mortar samples of similar dimensions to those required can be extracted from oversized vertical joints of the masonry, where the mortar is, unfortunately, characterized by noticeably lower compaction. Alternatively, the samples of dimensions close to 4 × 4 × 4 cm^3^ can be obtained by gluing together three samples (of dimensions 4 × 4 cm^2^ in the plane) cut from the bed joint (average 13 mm thick) [7]. However, the results obtained by this method are understated [8]. Currently, no general European standard contains a consistent methodology for determining the compressive strength of the mortar based on in situ tests, which is a very undesirable state.

The aim of this article is to evaluate the possibility of using three modern non-standard methods (PT, DPT and TPT) to estimate the compressive strength of the weak mortar in the bed joints of a brick wall. Additionally, an attempt was made to determine the possible limitations of each method by comparing the compressive strength established in all three tests with the reference compressive strength established as described in EN 1015-11 [6]. Moreover, performing tests several times on specimens of different ages (especially important are those results performed on specimens older than 4 weeks, which is the common total length of the research period in the majority of the articles) allowed for noticing the change in the relationship between the results.

The vast majority of non-destructive and minor-destructive tests are affected by an error due to the fact that the mechanical parameters of the material are not determined directly but by correlation. Extensive studies of this phenomenon were made in [9,10]. For example, for the PT test, it is the correlation between the hardness of the mortar and its compressive strength. The biggest advantage of minor-destructive methods is the ability to perform a large number of in situ tests, which is crucial in statistical evaluation. Especially with modern mortars based on recycled powder [11] or increased porosity [12]. In addition, the equipment needed to perform these tests is significantly cheaper compared to the machines used for destructive tests. Moreover, most of the MDT allows for immediate interpretation of results.

## 2. Minor-Destructive Testing Methods

The joint that has the greatest influence on the wall’s compressive strength and deformability is the bed joint, so all diagnostic tests are conducted on it. The mortar contained in the vertical joints is more porous and poorly reflects the load history of the structure, so it is not suitable as a testing material. Due to the insignificant thickness of bed joints (on average 10–20 mm), the number of diagnostic tests that may be applied is limited considerably. The tests used commonly in the diagnostics of the existing structures, such as the Schmidt hammer test or ultrasounds, cannot be applied here. Three methods were analyzed, which, in the opinion of this paper’s author, apart from the determined test methodology, are characteristic of their high potential in the diagnostics of historical structures with exceptional value.

### 2.1. Double Punch Test (DPT)

**Figure 1:**
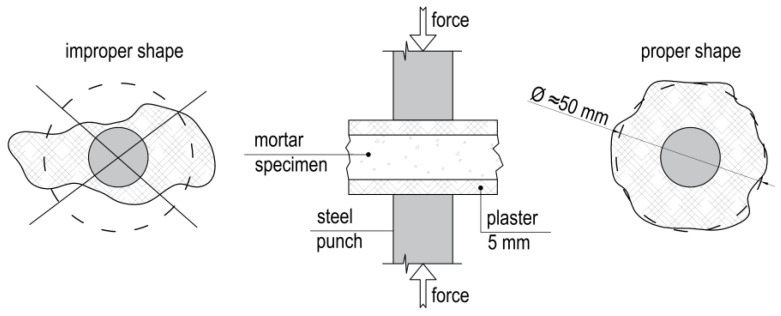
The idea of DPT—compression test through steel punches. Specimen suitable for test should have about 50 mm in diameter and both surfaces leveled.

The DPT was developed to test mortar samples of irregular shape and a thickness equal to that of the bed joint from which the specimens were taken [13]. The detailed methodology of the performance of the test is contained in DIN 18555-9 [14] and the UIC 778-3 instruction [15]. The test specimens are usually obtained from fragments of masonry characterized by a weak bond between brick and mortar or by drilling from the wall cylindrical specimens (50 mm diameter) with a centrally placed bed joint. Next, the mortar layer was separated from the bricks with a chisel. Specimens with a radius in the projection of about 40–50 mm qualified for testing. The upper and lower surface of the specimen is leveled with a material of similar strength to the tested mortar—most often, a layer of plaster with a total thickness of 5 mm is used. The test involves applying a compressive force through steel punches (Figure 1) until the destruction of the specimen.

The main advantage of this test is the direct measurement of the mortar’s mechanical properties. A negative feature of this test is the high susceptibility of its result to a range of factors, such as specimen slenderness [16,17,18,19], a manner in which the surface is leveled [20,21], the shape and diameter of steel punches [16] or the presence of large aggregate grains [22]. In addition, in the case of walls with good adhesiveness between brick and mortar, extracting specimens is really difficult or even impossible. The number of correlation functions between mortar strength acc. to DPT test (*f_m.DPT_*) and mortar reference strength (*f_m_*) is very limited, and some attempts were made in [16,22,23].

### 2.2. Standard Penetrometric Test (PT)

**Figure 2:**
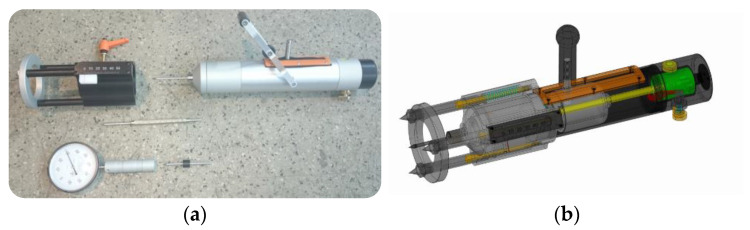
Impact penetrometer RSM-15: (**a**) the penetrometer full kit; (**b**) the device mechanism [29].

The general idea of penetrometric tests is to record the depth at which the needle penetrates the bed joint as a result of successive impacts. The impact force is generated by a mass connected to a releasing spring—a mechanism similar to one in a Schmidt hammer. In practice, a few types of penetrometers were used [24,25,26,27,28], but most of them were only in laboratory tests. This article uses the first serially manufactured modern penetrometer, RSM-15, with a standard impact energy of 4.55 Nm and needle diameter of 4.5 mm (Figure 2).

Contrary to the still-only prototype version of the static penetrometer [27], the impact version of the penetrometer [29] is simple and quick to use. Similarly to the Schmidt Hammer, which is popularly used in concrete diagnostics, the most important is the proper selection of the test place so that the recorded values reflect the real technical condition of the tested wall fragment. Due to the lack of information about the influence of high moisture content in a wall on results, all tested areas should be identified earlier using the available NDT methods such as thermography or gravimetric method [30]. The main advantages of the PT test are as follows: low invasiveness, fastness, the possibility of measuring at the specific depth of the joint and the availability of the result directly after finishing the test. A basic disadvantage is a small database of the published results of experimental tests, which would enable us to determine what factors and to what extent affect the result. Furthermore, strength is defined indirectly with the use of a correlation curve.

According to the manufacturer (Diagnostic Research Company—DRC) [29], the device allows to make:

- A measurement of the PT homogeneity of the mortar between its successive layers in terms of degradation, carbonation or material heterogeneity;
- An assessment of whether the entire structure has been erected using the same mortar;
- An assessment of the compressive strength of the mortar.

There are several methods [25,31,32,33] similar to PT that are currently being used for mortar diagnostic, e.g., “Sphere impact”, “controlled penetration” or “DRMS” (based on microdrilling resistance technique and is a modification of older PNT-G test [34]). Moreover, new methods, such as the “gun penetrometer”, are still under development [28]. 

### 2.3. Torque Penetrometric Test (TPT)

**Figure 3:**
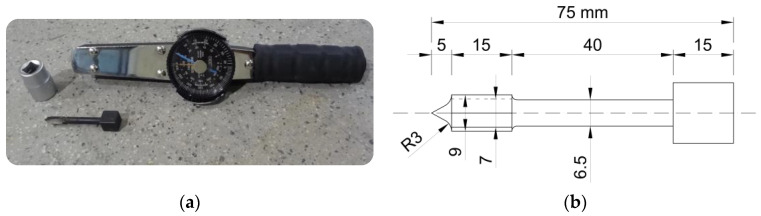
Torque penetrometer: (**a**) complete testing kit used in this article; (**b**) measurements of penetrating nail.

The TPT method applied in the article was proposed by D. Marastoni [35,36] in 2016 as a modification of the X-Drill method [37], together with the solutions known from the geotechnical Vane Shear Test [38]. The newly developed method is based on the correlation between mortar compressive strength and the value of torque needed to cut off the tested mortar by the toothed part of the penetrating nail (Figure 3b). The complete testing equipment consists of a penetrating nail and a torque wrench (Figure 3a). In addition, it is necessary to use a drilling machine and a hammer in order to insert the penetrating nail into the examined mortar.

Because the diameter of the penetrating nail equals 9 mm, one should use bed joints more than 11 mm thick to avoid scratching the toothed part of the penetrating nail against the bricks, which determines the masonry joist dimensions. The pros and cons of the TPT test are similar to the PT test. A well-known method similar to TPT is the screw (helix) pull-out method [39].

## 3. Research Methodology

**Figure 4:**
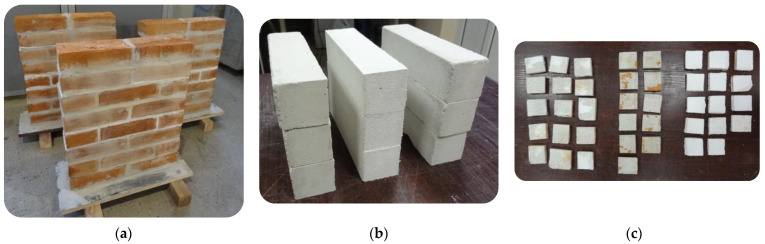
Tested elements: (**a**) brick masonry prisms; (**b**) standard specimens 4 × 4 × 16 cm^3^; (**c**) DPT specimens ≈ 5 × 5 cm^2^.

In order to estimate the range of applicability for the DPT, PT and TPT methods, 3 brick-masonry prisms were built, with dimensions of 51 × 12 × 62 cm^3^ each (Figure 4a). There were 12 × 25 × 6.5 cm^3^ bricks used for the prisms. All the bed joints had a fixed constant thickness because of the use of 12 mm spacer strips, which is of particular significance in the case of TPT testing.

Each of the three MDT tests was carried out on three types of mortar (two of them were cement–lime and one was lime) in order to achieve a greater comparative database of all the results (Figure 4b). 

In order to replicate the real conditions in which historical buildings were erected, it was assumed that the components of the mortar would be dispensed by volume—so-called “prescribed mortars”. The ratio values were assumed according to PN-B-10104 [40] code for F, G and H mortar (Table 1). In every prism, one bed joint was specifically prepared to allow for the extraction of specimens for the DPT test (Figure 4c). Applying a separating layer in the form of thin filter paper helped weaken mortar adhesion to the brick. In addition, brick surfaces in this joint were smoothed by grinding. For each of the three types of mortar, three samples 4 × 4 × 16 cm^3^ were prepared to determine mechanical parameters according to EN 1015-11 standard [6]. After 7 days of maturing in high air humidity, the prepared specimens were placed in the same room as the masonry prisms in order to ensure comparable climatic conditions.

**Figure 5:**
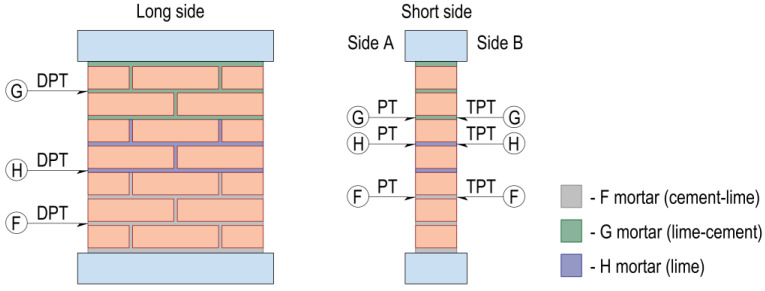
Construction of brick masonry prisms prepared for testing. All bed joists were tested. Three joists were dedicated to the DPT tests. The PT tests were conducted on the remaining bed joints on one side (A) of the masonry prism. The same bed joints, but on the opposite side (B) of the prism, were intended for the TPT tests.

The MDT tests were thoroughly comparable due to the fact that each of the masonry prisms was layered with bricks with the use of all three mortars, F, G and H. Additionally, this allowed for increasing the size of the results database. The detailed structure of masonry prisms and the location of the respective MDT tests is presented in Figure 5. During PT and TPT tests, each of the examined prisms was subject to compression of 0.15 MPa in order to immobilize it.

**Figure 6:**
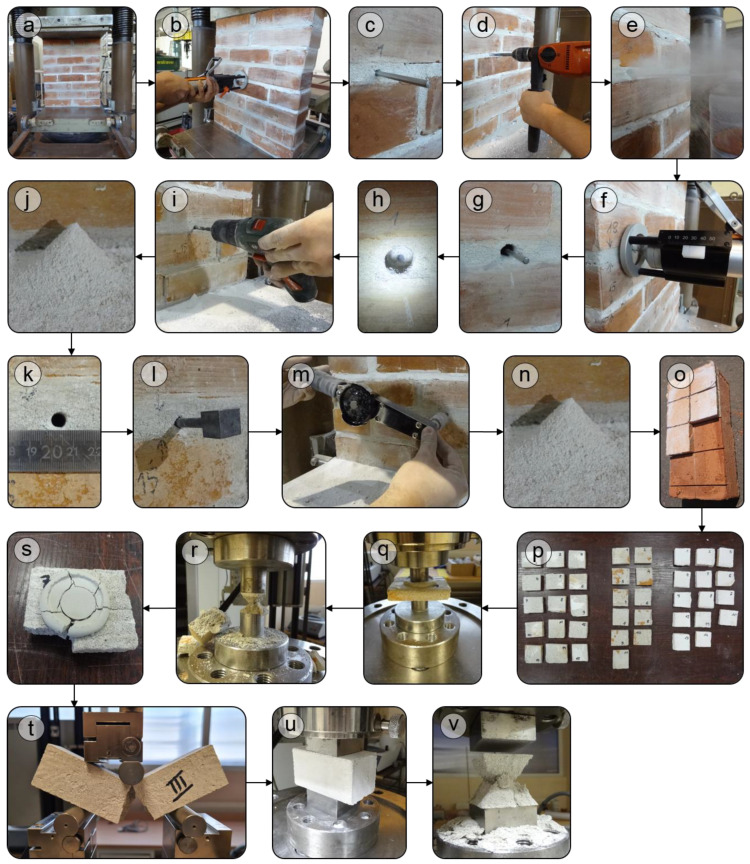
The sequence of the tests: (**a**) the immobilisation of the prism with the force generating the pressure of 0.15 MPa; (**b**) the performance of the PT test at the outer surface; (**c**) the verification of the correctness of the needle settlement in the middle of the joint thickness and its horizontal position; (**d**) the immersion drill hole with the diameter of 12 mm at the depth of 4 cm; (**e**) the removal of dust with compressed air; (**f**) the performance of the downhole measurements during PT test; (**g**,**h**) the verification of the correctness of the needle settlement as in “**c**”; (**i**) drilling at the depth of 5 cm of the pilot hole with the diameter of 7 mm; (**j**) the verification of the drill cuttings for the purpose of determining whether the brick layer remained intact or not; (**k**) the measurement of the diameter to ensure whether the probe teeth are anchored correctly or not; (**l**) sticking the probe in; (**m**) the performance of the TPT test; (**n**) the verification of the drill cuttings as in “**j**”; (**o**) when the PT and the DPT tests are completed, dismantling the brick prism and extracting the specimens for the DPT tests; (**p**) levelling the specimen surface for the DPT tests; (**q**) the DPT test; (**r**) the destruction form of the DPT specimen; (**s**) the crack pattern during destruction as the assessment of the lack of the eccentricity of the compressing force; (**t**) the performance of flexural tests on the referential specimens; (**u**) the performance of compression tests on the referential specimens; (**v**) the destruction form of the referential specimen during compression.

The PT test was carried out on the outer mortar surface (Figure 6b) and as the mortar downhole measurements (Figure 6f). Since the prisms for testing were prepared in the laboratory and not sampled from the existing structure, it was not necessary to remove the outer mortar surface, which may be corroded. At the first PT test stage, there were 20 strokes performed, and the needle probe immersion was recorded after the 5th, 10th, 15th and 20th strokes. As part of the second stage, there was a drill hole made at the same point, with a diameter of 12 mm and a depth of 4 cm in order to conduct the PT test on the mortar located inside the joint (Figure 6d). The drilling dust was removed from the drill hole by means of compressed air (Figure 6e). The reading of the needle probe immersion was re-performed after 20 strokes. By using the immersion depth of the needle probe after the 10th stroke, the mortar compressive strength was determined based on the correlation curve, DRC [29], which was referred to in the author’s previous paper ([22], Figure 6). On the basis of increments between 5, 10, 15 and 20 strokes the mortar homogeneity was assessed. The methodology was repeated in at least three points, located at a minimum of 10 cm from each other, for each of the tested joints. 

The TPT test was performed with the tension wrench, BAHCO (Figure 3a), with a measurement range up to 35 Nm and a reading accuracy of max. 0.5 Nm. In the first stage of the test, a pilot hole was made with a diameter of 7 mm and a depth of 5 cm. The drill cuttings obtained were checked in terms of the brick particle content, which would disqualify the drill hole from further tests due to the contact of the penetrating nail with the bricks, which are stronger than the mortar. The drill hole diameter was checked, and it was ensured whether, within the entire depth, there were any hollows or large aggregate grains that would distort the results obtained. The drilling dust was removed from the drill hole by means of compressed air.

The DPT test was performed after the completed PT and TPT tests. In order to extract the mortar specimens from three joints for the DPT tests, the subsequent layers of bricks were loosened. The joint-forming mortar was cut into specimens already at the stage of its joining with a brick in order to obtain the specimens with the intact structure as far as possible. Mortar specimen extraction was carried out with the use of a broad chisel. In order to ensure the flat surface of the specimen, it was leveled on both sides with a layer of fast-binding plaster. The leveling layer was obtained by means of steel punches with a diameter of 30 mm, at the same time ensuring the possible correction of the specimen position during the DPT test. For the regular DPT test, steel punches with a diameter of 20 mm were used (Figure 6q). The tests were performed on the testing machine, ZwickRoell AG (0.5 accuracy class of testing machine with an uncertainty of measurement 0.12%). The load increment in time was selected individually for each of the three mortar types for the specimen destruction between the 2nd and 3rd minute of the test. 

The tests of the standard specimens, 4 × 4 × 16 cm^3^, were conducted in accordance with the methodology described in EN 1015-11 [6]. In the first stage, flexural strength was tested (Figure 6t). The obtained specimen halves, with the dimensions of 4 × 4 × 8 cm^3^, were subject to compressive strength tests (Figure 6u). The tests were carried out with the use of the same testing machine as in the DPT tests. 

Repeating the minimally invasive test a few times on the same material is to minimize the possible unfavorable impact of local effects, which may be revealed during the test. Additionally, it increases the number of test results, which enables statistical analysis.

## 4. Results

### 4.1. The Mortar Reference Strength Acc. to EN 1015-11 [6]

As a result of the flexural tests and then the compressive tests performed on the mortars in accordance with the procedure provided in EN 1015-11 [6], the mortar mechanical parameters were determined after 4, 12 and 90 weeks. The figures are presented in Table 2 and Table 3.

**Figure 7:**
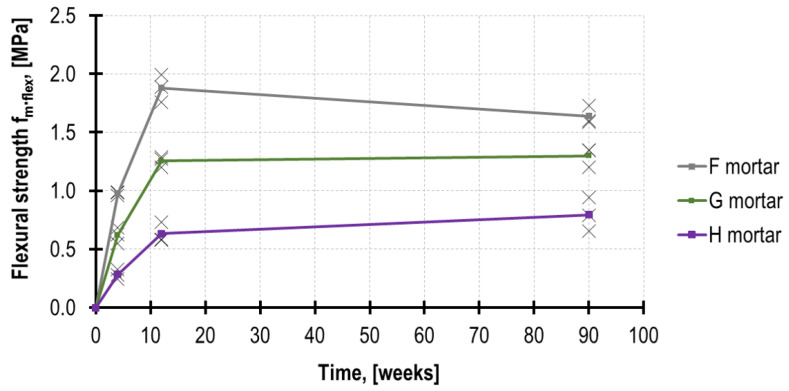
Flexural strength development in time.

**Figure 8:**
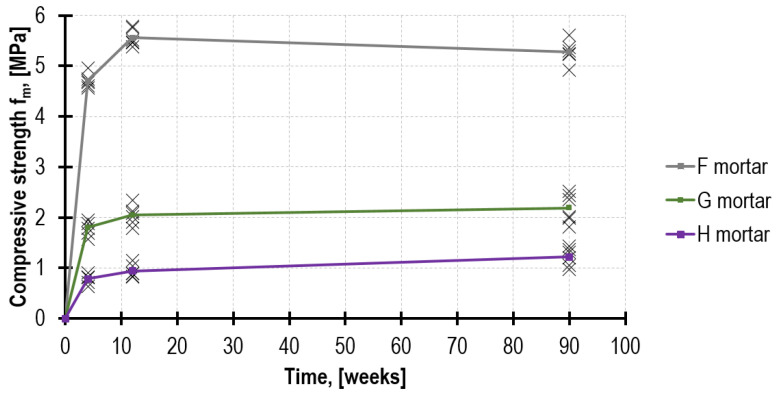
Compressive strength development in time.

As expected, after 4 weeks, the highest mechanical parameters were demonstrated by mortar F, which obtained *f_m_* = 4.71 MPa, whereas mortar H obtained merely *f_m_* = 0.79 MPa, which is only 17% in relation to mortar F. After 90 weeks, both tested parameters were higher; nevertheless, the compressive strength in the lime mortar (H) increased more intensely than in the remaining mortars, reaching the value of *f_m_* = 1.22 MPa. Furthermore, the results of the tests performed on mortar H specimens were characterized by higher values of variation coefficients, reaching 15%. In order to compare the strength development rate of the analyzed mortars in time, the recorded results are presented in Figure 7 and Figure 8.

When comparing the ratio of *f_m.flex_/f_m_* for respective mortars, after 90 weeks, its highest value was demonstrated by mortar H, reaching the value of 0.64, whereas mortar F had the lowest value—merely 0.31.

### 4.2. Mortar Strength Based on the DPT Test

The DPT test conducted simultaneously provided considerably higher values of the maximum compressive stress during the destruction of the specimen. This trend was noticeable for each of the tested mortars. The detailed results are presented in Table 4.

**Figure 9:**
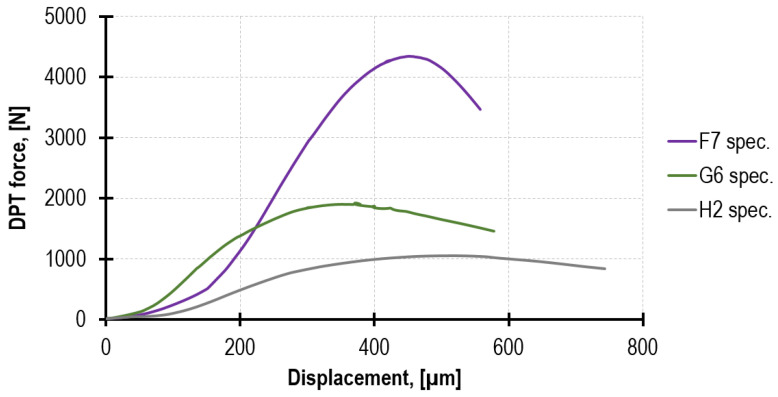
The comparison of the typical behavior of the three analyzed mortars, F, G and H, on the example of random specimens—the test performed after 12 weeks. (The displacement was recorded only by the machine grips; therefore, it involves a small distortion resulting from the elastic strain of the steel punches and the possible presence of backlashes in the machine).

The observed nature of the DPT specimen’s destruction was typical for this test [4]. As a result of the load increase, numerous cracks were formed, spreading radially from the edge of the compressive force application (Figure 6s). Their number and even distribution along the entire circumference excludes the existence of unintentional load eccentricity, at the same time proving the testing method to be valid. The mortar that remained between the steel punches had the form of a sandglass, which is typical for confined specimens (Figure 6r). Mortar F and G demonstrated more brittle destruction as compared to mortar H, for which the force close to the maximum was transferred in the wide range of steel punches displacements. Figure 9 presents the three diagrams of randomly selected specimens, representing the behavior of the tested mortars. 

**Figure 10:**
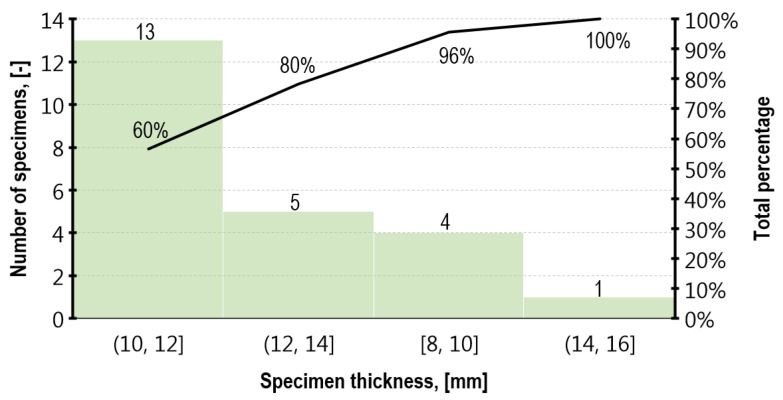
The percentage share of the respective DPT thickness values in the tested population after 90 weeks—no capping included.

When considering a strong dependence between *f_m.DPT_* and the specimen thickness, at the stage of prism masoning, brick spacers were used, thus obtaining similar specimens thickness. According to the layout presented in Figure 10, demonstrated on the tests performed after 90 weeks, as many as 80% of specimens fell within the range of 10 mm to 14 mm, which is typical for bed joints. As a result of leveling the top and bottom load surface with a plaster layer, the specimen’s average thickness was increased by 4.4 mm.

The highest values of various coefficients of 17% on average were recorded for lime mortar H, and twice lower, i.e., 8%, for lime–cement mortar G. What is typical here is the increase in the results dispersion of the tests conducted on mortar specimens with low strength, which were extracted from the structure. It appears that the type of binder used (lime; lime–cement; cement) is more significant here than the mortar strength resulting from such a binder.

### 4.3. Mortar Strength Based on the PT Test

**Figure 11:**
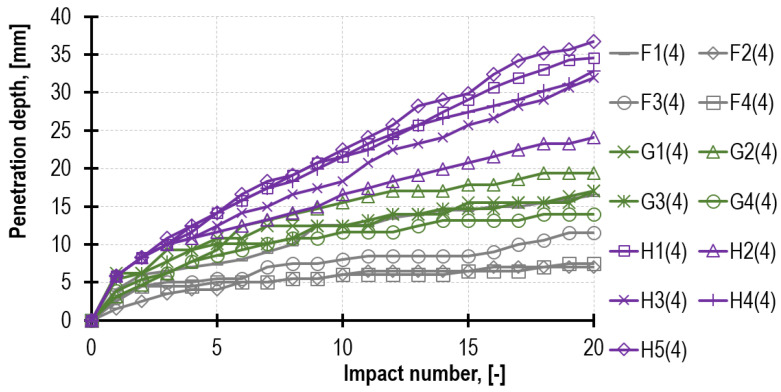
The results of the penetrometric tests conducted on the surface layer of mortar F, G and H after 4 weeks of the prisms masoning.

The penetrometric tests (PT) performed on three bed joints (of each of the prisms tested) demonstrated the mortar strength *f_m.PT_* closer to mortar reference strength *f_m_* (of the standard beam specimens) than strength *f_m.DPT_* determined directly from the test on the steel punches. This relation is also observed in other tests, not only the laboratory ones but those conducted on real structures [36,41]. The results from the downhole tests also refer to the mortar inside the joint, which is the material tested in the two remaining tests: DPT and TPT. The influence of the depth from which specimens are sampled from the joint may be significant [30,42,43]. The total number of readings of the needle probe immersion was 1240 (62 measurement series with 20 strokes each). Figure 11 presents the example of the penetration depth development curves recorded on the mortar surface layer after 4 weeks of the prisms masoning.

In order to compare the results, it is possible to introduce the notion of the needle probe “drive”, defined as the average value of the needle immersion per 1 stroke and calculated from the entire series of 20 strokes. The drive values calculated during the tests performed on the surface layer of mortar F, G and H after 4 weeks are as follows, appropriately: 0.63 mm, 0.89 mm and 1.60 mm. The same values, determined on the basis of the downhole tests (after performing the drill hole of 4 cm), amounted to, respectively, 0.50 mm (decrease of 21%), 0.81 mm (decrease of 8%) and 1.76 mm (increase of 10%). These results suggest that mortar mechanical parameters may differ depending on the depth at which the test is performed.

**Figure 12:**
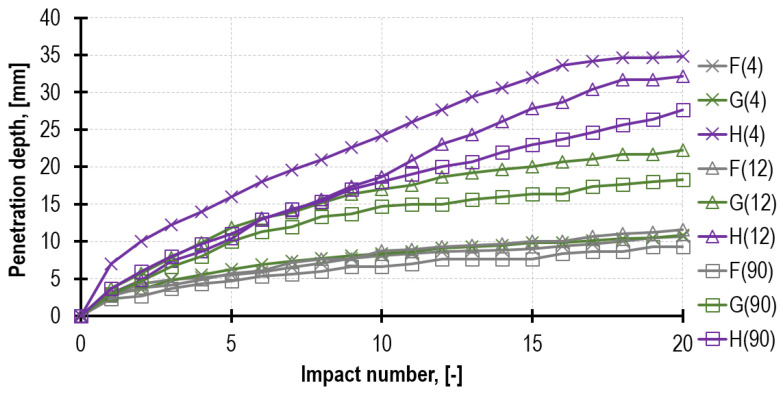
The averaged results of all the penetrometric tests (surface and downhole) for mortar F, G and H performed in 3 time intervals, after 4, 12 and 90 weeks.

By averaging the results to keep only the division due to the mortar type and its age as of testing, Figure 12 was obtained. As anticipated, the most advantageous results were obtained for mortar F, then for G and H. Regardless of the mortar type, as time passed, the parameters generally improved. The decrease in the total penetration depth between the 4th and 90th week was 4.7 mm (44%) for mortar F, 6.9 mm (41%) for mortar G and 21.8 mm (68%) for mortar H.

By converting the obtained penetration depths into the mortar compressive strength, with consideration of the division into mortar types and age, Table 5 was obtained. 

In most cases, the mortar strength estimated in the PT test increases as its age grows. Regarding mortar F with the prevailing cement share, 67% of the final strength was achieved after merely 4 weeks of maturing. In contrast, the lime mortar, at the same time, developed only 22% of its final strength.

**Figure 13:**
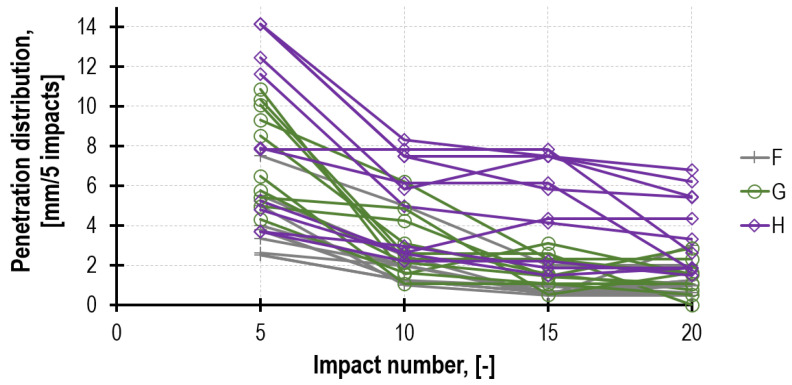
The penetration distribution divided into the mortar type demonstrated by outer surface measurements.

A penetration distribution diagram was used to assess whether there was a stabilization of the boundary conditions of the needle probe immersion during the test, which is presented in Figure 13 for outer surface readings.

From among 62 functions of penetration distribution, 71% achieved the stabilization of the needle immersion depth already in the second group of five strokes, which indicates the insignificant extent of boundary distortions. Only 12% of curves did not achieve a decrease exceeding 50% of the final needle immersion after 10 strokes.

### 4.4. Mortar Strength Based on the TPT Test

**Figure 14:**
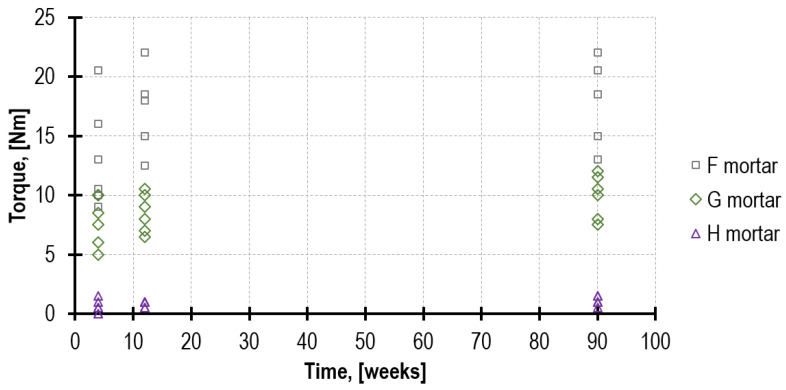
The torque values recorded for mortar F, G and H with the use of 7 mm pilot hole. The H mortar did not allow for the proper anchorage of the penetrating nail.

For mortars F and G, as the time passed by (4, 12 and 90 weeks), the value of the torque needed for shearing the mortar through the toothed part of the penetrating nail increased as well. The average torque values for mortar F amounted to 13.2 Nm, 16.8 Nm and 17.9 Nm. For mortar F, they amounted to 7.8 Nm, 8.5 Nm and 9.9 Nm. Regarding mortar H, the increase in the torque value in time was around the reading error, and it amounted to 0.6 Nm, 0.8 Nm and 1.1 Nm. Results were compared in Figure 14.

The mortar compressive strength was converted based on equation number 15 provided in [36]. The strength *f_m.TPT_* determined in the TPT test for mortar F and G obtained the values slightly exceeding reference strength *f_m_* (Table 6). Unfortunately, the insufficient anchoring of the penetrating nail in weak mortar H obtained values different than expected. As a control, in the 12th and 90th week, the tests were performed with the pilot hole diameter reduced from 7 mm to 4 mm. This improved the penetrating nail anchoring (increasing *f_m.TPT_* value six times); nevertheless, the results obtained had highly random nature, sometimes higher and sometimes lower than reference strength *f_m_*. The impact of the pilot hole diameter on the penetrating nail anchoring in the joint was also observed [44,45]. This phenomenon requires further tests. Due to those problems, TPT tests for H mortar were mostly omitted during the below analysis.

## 5. Discussion

The ratio of mortar reference strengths *f_m.flex_*/*f_m_* depends both on the binder amount and type and the age of the specimens tested. In the tests carried out on 4 weeks old specimens, the lowest ratio, equalling 0.21, was obtained for the strongest mortar, i.e., F, and the highest ratio, equalling 0.36, was observed for the weakest mortar, i.e., H. Similar values are acknowledged in the literature [40,42].

The specimens’ age also had a consistent impact in the case of all the mortar types. The strength ratio achieved the highest value for the tests performed in the 12th week (0.34 for F, 0.61 for G, 0.67 for H), and in the tests in the 90th week, there was a slight decrease (maximum 8% for mortar F containing the most cement). A few factors may have an impact here. The most important one is the difference in the strength increase dynamics *f_m.flex_* in relation to *f_m_*, and the second one is the development of micro cracks as a result of shrinkage. The shrinkage impact on the reduction in flexural strength grows along with the increase in the binder/aggregate ratio [42]. The slight decreases in the mortar strength over time were also recorded in publications [41,44], where this decrease was Δ*f_m.flex_* = −18% and Δ*f_m_* = −21%, respectively.

**Figure 15:**
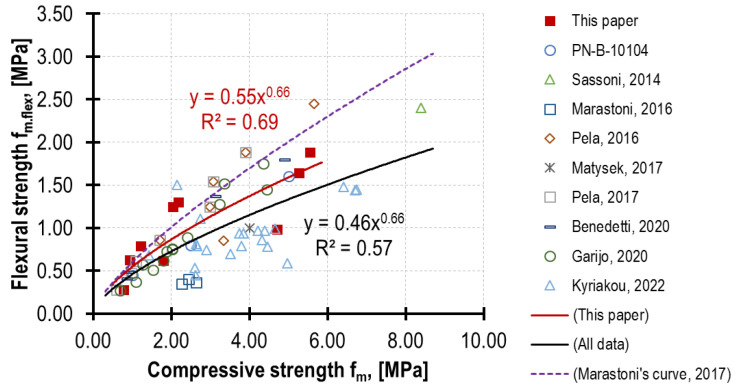
Correlation between mortar compressive strength and mortar flexural strength. Specimens prepared and tested according to EN 1015-11 [6]. Comparison between author’s test results and results from the literature [3,35,41,44,45,46,47,48].

The comparison of the author’s test results with the results of other researchers (Figure 15) demonstrates that the relationships of mechanical parameters depend greatly on the mortar composition and curing regime [42]; numerous correlations were published in [46]. The mortars tested in this paper are similar to mortars used typically in the existing brick structures—the obtained red curve (in Figure 15) falls within the dependence devised by Marastoni in [36], and the black curve describes all the included results. In almost all cases, flexural strength increased more rapidly than compressive strength; the same results were noticed in [46].

**Figure 16:**
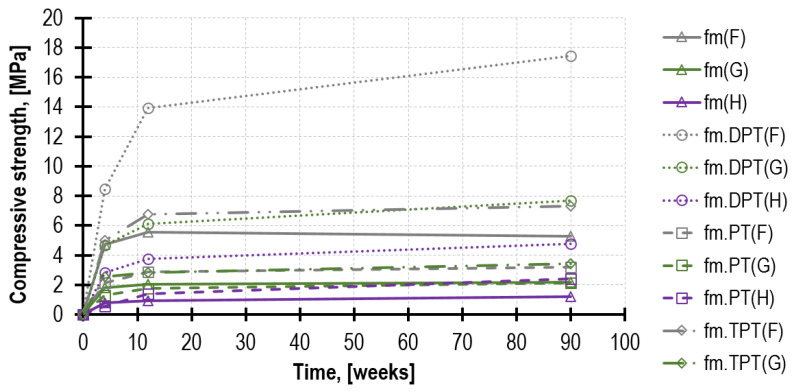
Mortar strength development in time according to different testing methods. The respective test types are marked with a given line style, and the respective mortar types are marked with a given color.

The obtained curves of the mortar compressive strength increase in time, depending significantly on the testing method (see Figure 16). The highest values were always obtained from the DPT (dotted line) tests, and the significantly lower ones were from the TPT (dash–dot line) tests (except for mortar H, for which no result was obtained). Moreover, the results of the PT tests (dashed line) and the tests performed on reference beam samples (solid line) provided the lowest strengths (*f_m_* for mortars H and F, and *f_m.PT_* for mortar G). Significantly higher values of *f_m.DPT_* result from a few factors, among which the most important are the following:

(a) Specimen confinement (the effect of friction at the specimen—punch contact zone; a specimen area larger than the load area; the presence of a capping) [16,20,47,49,50].
(b) Specimen small dimensions and slenderness (the ratio of specimen thickness to punch diameter) [8,16,17,22,26,51].
(c) The beneficial mortar curing regime inside the wall [20,52].

The impossibility of the penetrating nail anchoring in mortar H in the TPT test suggests excluding this method from use in the case of very weak mortars. This thesis is confirmed partially by the results obtained by this method’s author [36], where only one mortar type was tested with a strength of <1 MPa. The differences recorded between the readings were 30%. At the same time, the difference between the mortar strength, determined based on the correlation with the non-destructive test result, ranged from 31% to 80%.

**Figure 17:**
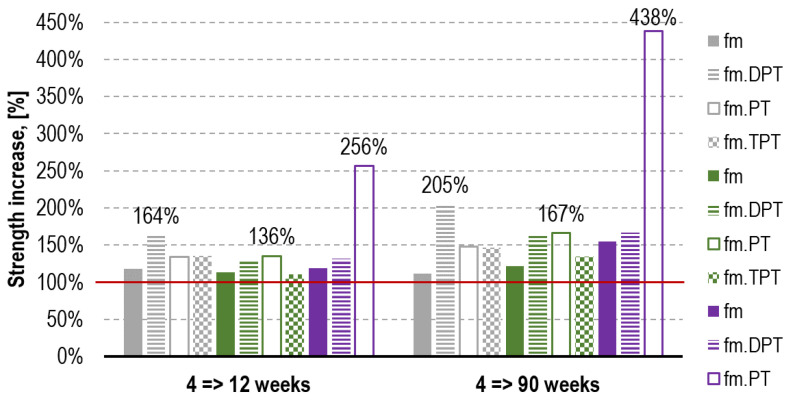
Mortar strength increase in time period between 4th and 12th week (e.g., *f_m_(12)*/*f_m_(4)*) and between 4th and 90th week (e.g., *f_m.DPT_(90)*/*f_m.DPT_(4)*). The red line represents the compressive strength established after 4 weeks of curing. The respective test types are marked with a given filling style, and the respective mortar types are marked with a given color.

In order to determine the impact of the mortar curing regime, it is necessary to analyze its strength increase dynamics for each of the tests carried out. Figure 17 presents the above as the comparison of the values between the 4th and the 12th week and the 4th and the 90th week. The highest relative increases (the increase from 0.55 MPa to 2.41 MPa, the ratio of 438%) are characteristic of the PT tests performed on mortar H. This effect probably arises from the highly advantageous coincidence of three factors. The first one, triggered by the presence of bricks, is the reduction in excessive water in the mortar mixture during hardening in the joint. This phenomenon, described in the literature as the “absorption effect”, has a significant impact on the increase in the mortar strength in the actual joints of the structure [28,53,54]. Furthermore, it contributes to the considerable reduction in the total open porosity and the reduction in the average pore radius [20,52]. The other factor is fast carbonation which starts in the mortar’s outer surface of the joint, which is where the PT tests [42] are performed. The third factor is the natural tendency of lime mortars for slow hardening [46,48]. To compare, the ratio of reference strength *f_m_* in this period for mortar H was merely 155% (the increase from 0.79 MPa to 1.22 MPa). Regarding mortar F with the prevailing cement share, the highest strength increase was observed for the DPT tests.

Figure 16 and Figure 17 and the decreases in the total penetration depth (provided in Section 4.3) in the PT test confirm a strong relationship between mortar strength and its resistance during needle probe penetration. The identical dependence was also confirmed in [25], where after 6 weeks of curing, the penetration depth was reduced by 15% (cement–lime mortar similar to G) to 21% (lime mortar similar to H). In a similar period (between the 4th and the 12th week), in the tests presented in this paper, the reduction was, respectively, 16% for mortar G and 32% for mortar H. A considerably weaker relationship was obtained in [28] between mortar compressive strength and the number of strokes.

**Figure 18:**
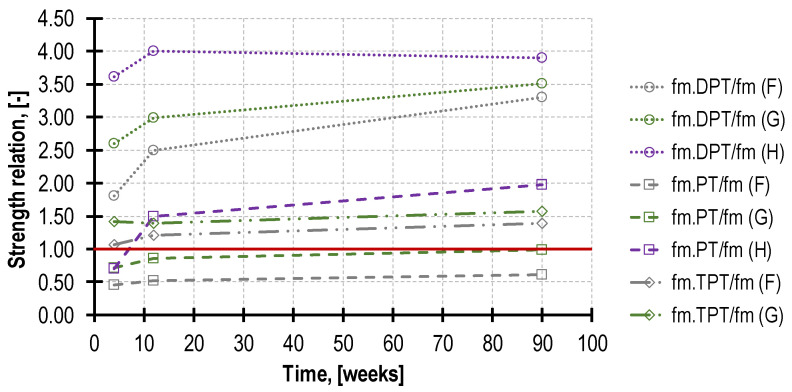
Time changing strengths relation, allowing to convert mortar strength obtained by all MDTs to reference strength *f_m_*. The red line separates MDTs that return mortar strength higher than *f_m_* (are above) from those which return values lower than *f_m_* (are below).

The minor-destructive test on the mortar in the existing structure is frequently performed in order to use the results obtained for determining the masonry compressive strength [55,56,57]. In such a case, it is often necessary to convert the estimated mortar strength obtained in the MDT test to the mortar compressive strength determined on half-beam specimens 4 × 4 × 8 cm^3^ [58]. The values of the conversion coefficients obtained in the tests for the respective MDTs in the time function are presented in Figure 18. Nevertheless, their application scope must be limited only to mortars with a similar composition and a testing methodology imitating the one adapted herein. Regarding the TPT and PT tests (with the exception of mortar H), the conversion coefficient value is nearly constant over time. As far as all the DPT tests are concerned (and PT for mortar H), the coefficient values demonstrate a significant increase over time. This is a consequence of the afore-described phenomena.

**Figure 19:**
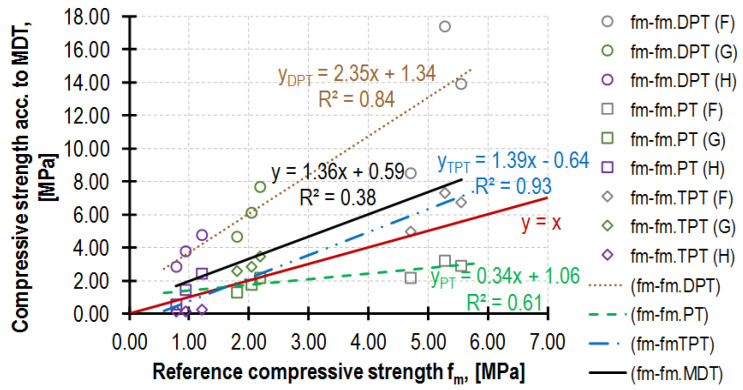
Comparison of mortar strengths obtained by MDTs (e.g., DPT, PT, TPT) and DT (test on 4 × 4 × 8 cm^3^ mortar half-beams). The red line separates MDTs that return mortar strength higher than *f_m_* (are above) from those which return values lower than *f_m_* (are below).

However, in most cases, the composition of the tested mortar is not known, which necessitates using a function with considerably lower accuracy yet a wider application scope. The dependences obtained in the tests *f_m.DPT_*(*f_m_*), *f_m.PT_*(*f_m_*) and *f_m.TPT_*(*f_m_*) are presented in Figure 19. Defining a single correlation curve for all three MDT tests (a black line in Figure 19) is irrational in practice due to the excessively large dispersion of results (R^2^ = 0.38).

**Figure 20:**
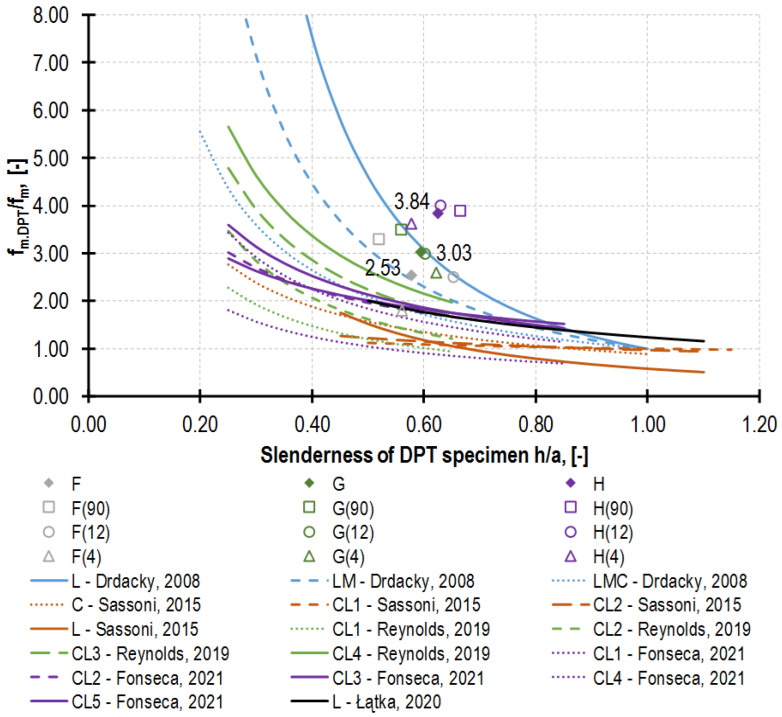
Relationship between specimen slenderness (specimen thickness, *h*/punch diameter, *a*) and value *f_m.DPT_*/*f_m_*. Comparison between author’s test results (marked by dots) and functions prepared based on other authors’ research (grouped by color of function) [16,18,19,20,22]. The interpretation of the symbols assigned to the binders used: L—lime; C—cement; LMC—lime–metakaolin–cement. The curves presented are not entirely comparable due to slight deviations in the testing procedure applied by the respective authors: [16] tests performed after 100 days of curing with the use of square punches with the side of 20 mm; [18,19] compressive strength was determined based on the ASTM C109 standard [59], i.e., on the cube with the side of 5 cm, understating it slightly. Slight difference in vertical location between author’s results and other functions seem to be due to lack of capping for DPT specimen in the case of majority of tests in the literature. Additionally, lower mortar DPT strength in the literature is caused by out-of-joint hardening of mortar (lacking favorable absorption effect).

Regarding the multiplicity of factors affecting the DPT test results (described in detail in the introduction to this section) and, in consequence, considerable variability of ratio *f_m.DPT_/f_m_*, it would be most advantageous to devise a few curves representing the types of mortars that are used most often. Since papers [16,18,19,20] provide the detailed results of strength *f_m.DPT_* and *f_m_*, as well as geometrical data for each tested specimen, the dependences between the DPT specimen slenderness and the correlation coefficient, could be elaborated by the author. Said dependences are presented in Figure 20, with consideration of a function, also elaborated by the author in [22], dedicated to the historical buildings of the City of Cracow (a black curve).

A continuous line marks the weakest mortar in a given series, a dotted line marks the strongest mortars, and a dashed line marks the intermediate-strength mortars. The strong curvilinear nature of the functions obtained confirms that the correlation coefficient value (*f_m.DPT_/f_m_*) increases as the specimen slenderness decreases (due to the increase in the specimen of the transverse compressive stresses in the radial direction). Another significant factor, which has not been emphasized sufficiently in the literature so far, is mortar strength. The weaker mortars were tested, and the higher correlation coefficient values were obtained. This is also confirmed by the results presented in the diagram in the form of dots recorded for the mortars analyzed herein. The following values of correlation coefficients were obtained for them: 2.53 for F, 3.03 for G and 3.84 for H, despite the comparable slenderness of the DPT specimens.

**Figure 21:**
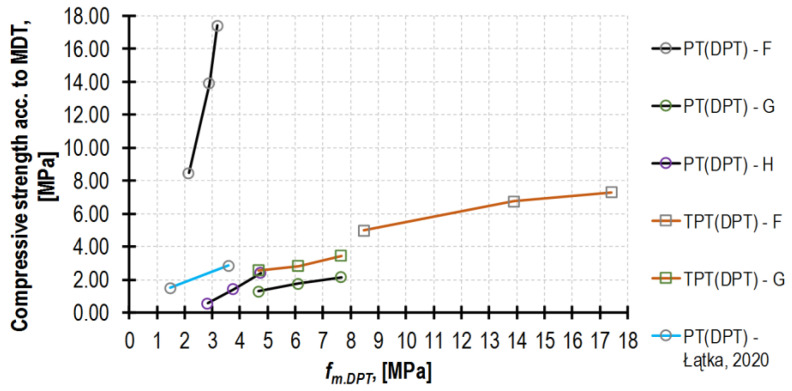
The correlations between the results of the MDTs and DPT. For comparison purposes, the correlation obtained from the author’s tests conducted on historical buildings [18] was marked with blue line.

The correlations between the respective MDTs and DPT tests may be presented successfully with linear functions (Figure 21). This feature was not dependent on the mortar type.

**Figure 22:**
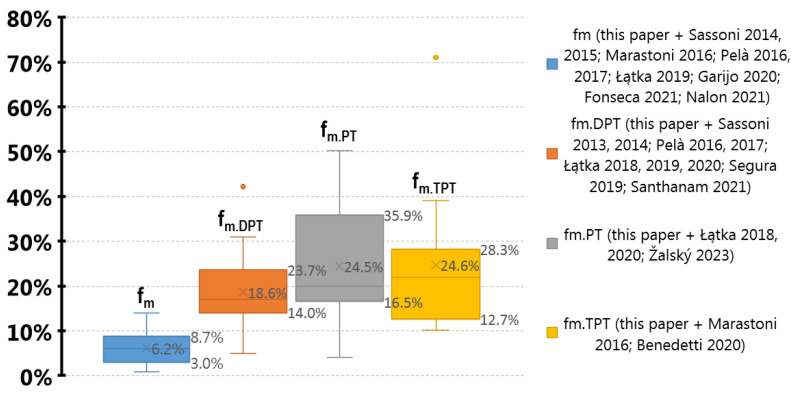
Comparison of variation coefficients (CV) for all analyzed tests from the literature [3,19,20,22,28,35,41,44,45,46,49,50,51,52,58,60,61] and presented herein. Total number of 140 CVs was included—64 for *f_m_*; 43 for *f_m.DPT_*; 19 for *f_m.PT_*; 14 for *f_m.TPT_*. The horizontal line represents median, and *x* represents mean. All MDTs are characterized by much wider dispersion in comparison with DT. The widest range of CV was noted for penetrometric test. The right skewed distribution characterized all CV tests.

By analyzing statistical parameters of all results presented in this paper and obtained by other authors [3,5,19,20,22,28,35,41,44,45,46,50,51,58,60,61], a significant difference between minor destructive tests (DPT, PT, TPT) and the destructive test is noticeable. For dispersion assessment, variation coefficients were compared in Figure 22. Only reference compressive strength results *f_m_* were characterized by a mean value of CV lower than 10% (precisely 6.2%). All the results of MDTs had this parameter 3–4 times higher. On the other hand, mortar reference strength was tested only on laboratory-prepared specimens with naturally high homogeneity. The value of CV seems to also be affected by mortar type. For all tests performed in this paper, the CV for the strongest mortar (F) was lower than for the weakest mortar (H). The mean value of CV changed from 3% to 12% for *f_m_*, from 11% to 15% for *f_m.DPT_*, from 12% to 26% for *f_m.PT_*, and from 28% to 58% for *f_m.TPT_*. This agrees with the conclusion that more homogenous mortars tend to be stronger [58]. Those two features suggest that in the case of low-strength mortar diagnostic with PT or TPT, a larger number of tests is needed for the same accuracy of estimation. According to [62], for practical applications, this dependency seems to become negligible for a number of NDTs measurements equal to at least 20.

## 6. Conclusions

In summary of the above results of the tests and analyses, the following general conclusions can be formulated:

- For the mortars tested, the weaker the mortar, the higher the strength ratio *f_m.flex_*/*f_m_*. The increased dynamics of compressive strength *f_m_* depends greatly on the binder amount and type; mortar F with a prevailing cement share, after 4 weeks, reached 89% of its final strength. For the mortars with a prevailing lime share, the higher the lime content was, the longer their strength was developed; mortar H, after 4 weeks, reached 64% of its final strength.
- Furthermore, the curing regime also strongly determined the increased dynamics of mortar strength. In the PT tests conducted on the external surface of the joint filled with mortar H, the values recorded were higher than in the mortar downhole readings. Most likely, this results from the free carbonation in combination with the absorption effect initiated by bricks. For this reason, regarding lime mortars, in order to determine more effectively the average mortar compressive strength, the PT tests must be supplemented with downhole readings. In the event of the DPT tests, higher strength *f_m.DPT_* increases in time were registered for cement–lime mortar F, which may be connected with the beneficial mortar curing regime inside a masonry, where humidity conditions are stable.
- Mortars with extremely low strength (<1 MPa) may not guarantee the required anchoring of the penetrating nail in the TPT tests while significantly understating the results obtained. The diameter reduction in the pilot hole has a positive impact here; however, further research in this regard is needed.
- The mortar penetrometric tests (PT) performed in joints, on account of the insignificant impact energy of the device, are limited to weak mortars only. The minimum penetration depth after the 10th strike, which may be converted based on the correlation curve, is 4 mm. This corresponds to the maximum mortar strength of 3.2 MPa. Moreover, the maximum penetration depth is 20 mm, which provides the mortar strength of approx. 0.55 MPa. Due to these facts, the PT method application scope is relatively narrow, and it is limited mostly to lime mortars.
- Ratio *f_m.DPT_*/*f_m_* increases as slenderness (*h/a*) decreases in the tested specimens due to the mortar confinement increase when loaded. Another essential parameter, accompanied by the increase in ratio *f_m.DPT_*/*f_m_*, is the mortar strength decrease. According to the test results, the highest ratios of *f_m.DPT_*/*f_m_* are characteristic of weak lime mortars tested on the DPT specimens with small thicknesses. The application of simplification used commonly in engineering practice (*f_m_* = 0.5 ÷ 0.7 *f_m.DPT_*) may lead to the considerable overestimation of mortar compressive strength *f_m_*.
- The ratio of *f_m.PT_*/*f_m_* and *f_m.TPT_*/*f_m_* increased in the tests as the specimens’ age increased and as the mortar strength decreased.
- For the purpose of improving the estimating accuracy of the mortar compressive strength in the existing structure, it is recommended to use at least two types of tests simultaneously. While comparing the values of the variability coefficients for the respective diagnostic methods, it is recommended to use the destructive and non-destructive tests simultaneously, thus considerably limiting the number of specimens required for destructive tests, as shown in [62]. Special attention is needed in case of low-strength mortar diagnostic using minor destructive tests. For those cases, an increased number of tests is recommended.
- If it is not possible to perform a few types of NDT tests, then the DPT test is the first choice test for strong mortars, which may be extracted from the masonry. As far as weak mortars or non-extractable mortars are concerned, the PT test is recommended, and the TPT test is recommended for medium-strength mortars.

## Figures and Tables

**Table 1:** Mortar volumetric composition ^1^. Binders: CEM I 32,5 R + hydrated lime.

Type	Name	Cement	Lime	Sand
F	cement–lime	1	1	6
G	lime–cement	1	2	9
H	lime	0	1	1.5

^1^ Mean weight of 1000 mL of: cement ≥ 1.10 kg; lime ≥ 0.52 kg; sand ≥ 1.56 kg.

**Table 2:** Mortar flexural strength *f_m.flex_* in MPa.

Type	Total Number of Specimens	After 4 Weeks ^1^	After 12 Weeks ^1^	After 90 Weeks ^1^
F	9 [3/3/3]	0.98 (0.01/1)	1.88 (0.09/5)	1.64 (0.06/4)
G	9 [3/3/3]	0.62 (0.05/9)	1.25 (0.04/3)	1.30 (0.07/5)
H	9 [3/3/3]	0.28 (0.03/11)	0.63 (0.07/11)	0.79 (0.12/15)

^1^ In brackets, there are provided, respectively, standard deviation (MPa)/variation coefficient (%).

**Table 3:** Mortar compressive strength *f_m_* in MPa.

Type	Total Number of Specimens	After 4 Weeks ^1^	After 12 Weeks ^1^	After 90 Weeks ^1^
F	18 [6/6/6]	4.71 (0.13/3)	5.56 (0.15/3)	5.28 (0.21/4)
G	18 [6/6/6]	1.80 (0.13/7)	2.04 (0.18/9)	2.19 (0.26/12)
H	18 [6/6/6]	0.79 (0.09/11)	0.94 (0.12/12)	1.22 (0.17/14)

^1^ In brackets, there are provided, respectively, standard deviation (MPa)/variation coefficient (%).

**Table 4:** Mortar DPT strength *f_m.DPT_* in MPa.

Type	Total Number of Tests	After 4 Weeks ^1^	After 12 Weeks ^1^	After 90 Weeks ^1^
F	24 [8/8/8]	8.48 (0.93/11)	13.90 (1.09/8)	17.42 (2.41/14)
G	24 [8/8/8]	4.68 (0.42/9)	6.12 (0.34/5)	7.66 (0.71/9)
H	20 [6/7/7]	2.84 (0.48/17)	3.76 (0.53/14)	4.75 (0.65/14)

^1^ In brackets, there are provided, respectively, standard deviation (MPa)/variation coefficient (%).

**Table 5:** Mortar PT strength *f_m.PT_* in MPa—an average value from the surface and downhole measurements (after deepening the hole with a drill).

Type	Total Number of Tests ^1^	After 4 Weeks ^2^	After 12 Weeks ^2^	After 90 Weeks ^2^
F	20 [8/6/6]	2.15 (0.51/24)	2.89 (0.10/4)	3.19 (0.23/7)
G	20 [8/6/6]	1.29 (0.18/14)	1.75 (0.34/20)	2.15 (0.38/18)
H	22 [10/6/6]	0.55 (0.20/36)	1.41 (0.51/36)	2.41(0.15/6)

^1^ In each test point, 2 tests were performed: surface and downhole. ^2^ In brackets, there are provided, respectively, standard deviation (MPa)/variation coefficient (%).

**Table 6:** Mortar TPT strength *f_m.TPT_* in MPa.

Type ^1^	Total Number of Tests	After 4 Weeks ^2^	After 12 Weeks ^2^	After 90 Weeks ^2^
F (7)	18 [6/6/6]	4.99 (1.95/39)	6.75 (1.57/23)	7.31 (1.57/22)
G (7)	18 [6/6/6]	2.56 (0.77/30)	2.83 (0.62/22)	3.44 (0.73/21)
H (7)	18 [6/6/6]	0.11 (0.11/101)	0.15 (0.05/34)	0.21(0.08/39)
H (4)	8 [0/4/4]	-	0.64 (0.30/46)	1.40(0.46/33)

^1^ In brackets, there is the pilot hole diameter provided in mm. ^2^ In brackets, there are provided, respectively, standard deviation (MPa)/variation coefficient (%).

## Data Availability

Not applicable.
